# Self-directed teaching: a holistic framework for educator autonomy

**DOI:** 10.3389/fmed.2024.1479885

**Published:** 2024-11-20

**Authors:** Changiz Mohiyeddini

**Affiliations:** Oakland University William Beaumont School of Medicine, Rochester, MI, United States

**Keywords:** self-directed teaching (SDT), professional development, autonomy, reflective practice, lifelong learning

## Abstract

Over recent decades, the complexity of higher education in general, and teaching specifically, has increased significantly, resulting in a myriad of challenges for educators. Traditional approaches to teaching often rely on standardized curricula and top-down instructional methods. Therefore, they are critically scrutinized for their lack of adaptability and limitations in addressing the diverse needs of contemporary educators and learners. The purpose of this paper is to introduce the concept of self-directed teaching (SDT) as a response to the contemporary challenges in education and explore its relevance and potential impact on educators and learners. SDT is proposed as a holistic, theory-based, proactive approach that integrates multiple core aspects of the teaching process into a cohesive framework. It aims to empower educators to embrace their autonomy, control their professional development, and adapt their teaching strategies, much like the concept of self-directed learning (SDL) applies to students. SDT has the potential to promote educator autonomy, provide strategies to address burnout, and offer adaptable approaches to meet diverse educational contexts. It encourages educators to tailor their teaching strategies and engage in continuous professional development, positioning them to respond flexibly to changing educational demands. Furthermore, this article outlines the theoretical foundations of SDT, grounded in theories such as self-directed learning, self-determination theory, and constructivist theory. Key components of SDT including autonomy awareness, teaching needs diagnosis, goal setting, resource identification, and continuous evaluation and reflection are discussed including strategies for successful implementation of SDT.

## 1 Introduction

Teaching is challenging. Over recent decades, the foundation of higher education has changed dramatically. The tides of massification, marketization, globalization, and digitalization are continuously reshaping higher education and add to the complexity of teaching (1). Consequently, policymakers and higher education community regularly communicate new goals and expectations from educators driven by policy changes, financial constraints and the growing recognition of the fundamental value of diversity (2).

Perhaps the most pressing concern pertains to how to maintain and improve the quality of teaching amid continuous change and an ever-widening uncertainty (3–6). Traditional approaches to teaching often rely on standardized curricula and top-down instructional methods. Therefore, they are critically scrutinized for their lack of adaptability and limitations in addressing the diverse needs of contemporary educators and learners (7, 8).

Educators (henceforth used interchangeably with teachers) are expected to impart knowledge while preparing students for a future where critical thinking, life-long learning, and adaptability are highly expected and appreciated (9). However, on one hand, institutions are forced to mandate standardized curricula and assessment methods to ensure accountability. Yet, on the other hand, educators are expected to tailor their teaching approaches to personalize learning experiences to meet the needs of increasingly diverse student populations from different cultural, socio-economic, and linguistic backgrounds (10). This diversity, while fundamentally desirable and vital for human culture, introduces a range of learning styles, needs, and expectations that educators must address to ensure equitable learning opportunities for all students (11), creating a conflict between the demands for standardization and the necessity for personalization (12, 13).

At the same time, the growing reliance on institutional metrics like performance evaluations—that tend to focus on quantifiable aspects of performance (e.g., number of publications)—can devalue other important aspects of teaching, like fostering critical thinking or creativity. This can pressurize educators to prioritize activities that are rewarded by performance evaluations and undermine educators’ professional autonomy, leading to job stress (14) and dissatisfaction (15, 16). For instance, educators are required to excel in both teaching and research, yet the demands of research often detract from the time and energy available for teaching, potentially leading to a decline in teaching quality (17, 18).

Emerging technologies, such as artificial intelligence, augmented reality, virtual reality, and online learning platforms significantly impact how teaching is expected to be delivered to and consumed by students. The rise of digital technologies was significantly accelerated by the COVID-19 pandemic which forced academic institutions to shift to virtual learning. Irrespective of whether online platforms were suitable for their specific teaching content or individual tech-savviness, educators were forced to quickly adapt to new technologies and integrate them effectively, regardless of their existing skills, long-established teaching habits, or carefully crafted approaches developed over many years (19, 20). Even when the decision to incorporate digital tools or certain pedagogical approaches is mandated, the implementation of these methods often encounters resistance from traditional practices and reluctance to change by the same institution that mandated them in the first place (8, 84).

In addition, and ironically, while educators are expected to exercise academic freedom, they also face pressures to align their teaching and research with political, social, and institutional norms, which can compromise their sense of professional freedom adding to the complexity of their roles (12, 21–23).

These conflicting expectations create a challenging environment for educators facing growing pressure to develop innovative teaching approaches, which, of course, requires continuous professional development to stay current with the latest pedagogical strategies, technological tools, and content knowledge (24–26). While maintaining and gaining competence is undoubtedly a professional responsibility for educators, traditional professional development programs—often consisting of periodic workshops or training sessions—may not suffice to provide appropriate professional development (27). These programs are, by nature, generic and disconnected from the specific needs and working environment of individual educators (28). Given these challenges, it’s no surprise that there is a growing recognition that professional development should be ongoing, personalized, and seamlessly integrated into the daily practice of teaching to be more attainable, efficient, and applicable (29, 30). Thus, there is an urgent need for a new approach that supports educators’ autonomy, encourages them to take control of their professional development, tailor their teaching strategies and enables them to adapt to emerging challenges to meet their own diverse needs and those of their students (82, 83).

Therefore, the aim of this paper is to introduce self-directed teaching (SDT) as a novel, holistic, feasible, and theory-based framework designed to promote autonomy, intrinsic motivation, and adaptability. SDT enables educators to proactively define their teaching goals, diagnose their needs, identify development resources, and evaluate teaching outcomes.

Accordingly, the primary objective of this manuscript is to conceptualize SDT and examine its theoretical foundations. In doing so, it seeks to fill a critical gap in the literature by offering a framework for teacher autonomy that parallels the well-established concept of self-directed learning (SDL) for students. Furthermore, key components of SDT will be discussed, including autonomy awareness, teaching needs diagnosis, goal setting and attainment, resource identification, continuous evaluation, and self-reflection, offering practical guidance for educators to implement this approach. In addition, the paper will demonstrate how SDT addresses the challenges outlined in the introduction, such as maintaining teaching quality amid continuous change, balancing standardization with personalization, integrating emerging technologies, and mitigating the erosion of professional autonomy.

## 2 Definition of self-directed teaching

In the following, built upon the foundation of autonomy and intrinsic motivation, self-directed teaching (SDT) is proposed as a novel holistic approach in which educators recognize their autonomy to define their teaching goals and approach, determining their teaching-related needs, identifying resources for professional development, and evaluate the outcomes of their teaching practices. This approach emphasizes the dynamic interplay between the proactive pursuit of teaching goals and the adaptive adjustment of those goals in response to contextual constraints and available resources.

At the core of the proposed SDT framework is its potential to offer both stability and flexibility within a fast-changing educational environment. While these concepts might seem at odds, SDT envisions them as complementary. SDT can provide stability by establishing autonomy, self-motivation, and continuous professional growth as core principles giving educators a reliable structure to set goals, reflect on their practice, and pursue professional development (31). At the same time, it encourages flexibility by allowing educators to adapt their teaching strategies and objectives in response to emerging educational demands and the diverse needs of their students. This balance of stability and flexibility could empower educators to maintain consistency in their professional growth while remaining responsive and innovative in their teaching practices and also contributes to a more meaningful and fulfilling teaching experience (32).

Obviously, SDT extends beyond the acquisition of teaching skills and seeks to encompass a broader, more active role in shaping and refining an educator’s approach to teaching. Simply put, a self-directed approach to teaching suggests that, before educators ask themselves, “How do I want to teach?”, they should first consider, “What kind of educator do I wish to be?” (80). This prompts a reflection on whether they aspire to be an educator who exercises autonomy, acknowledges their responsibility for the quality of their teaching, or one who are directed by external mandates without a deep sense of ownership over their teaching practice.

Reeve and Su (33), p. 351 highlight that “Rather than thinking of teachers’ goals and sense of efficacy as stable and enduring characteristics, both are better conceptualized as developmentally fragile.” This suggests that the definition of “efficacy”—or related constructs such as “a good teacher”—is not fixed, but can shift over time, making it necessary for educators to revisit and reevaluate them. This notion supports the concept of SDT, which emphasizes the educator’s autonomy in developing and refining their teaching goals, skills, and need for professional development. This understanding of teaching as self-directed challenges the conventional belief that extensive teaching experience alone suffices to become a good teacher. An experienced teacher is not necessarily better equipped to adapt to a rapidly changing educational environment and successfully respond to conflicting expectations. While experience is valuable, it translates to skill when it has been subject to individual reflection (32, 34, 35).

## 3 Theoretical foundations of self-directed teaching

Several well-established educational theories, such as self-directed learning, self-determination theory, constructivist theory provide robust foundations to elaborate on SDT.

Self-directed learning (SDL) addresses the autonomy of learners, encouraging them to take initiative in determining their learning needs, setting goals, identifying resources, and evaluating their progress. In essence, SDL posits that adults are inherently self-directed learners capable of assessing, planning and executing their learning activities (36). Recent scholarly effort supports SDL’s relevance in fostering professional development of educators (37). Therefore, SDL principles directly apply to SDT. While SDL empowers learners to take control of their educational journey, SDT empowers educators to take charge of their teaching by establishing their teaching needs, setting feasible goals, and evaluating their progress, thus engaging in a continuous cycle of improvement.

As a fundamental tenant, self-determination theory (38) emphasizes the vital function of intrinsic motivation (motivation driven by internal rewards) and the psychological needs for autonomy (the need to feel in control of one’s own behaviors and goals), competence (the need to gain mastery of tasks and learn different skills), and relatedness (the need to feel a sense of belonging and attachment to others) for overall well-being and performance. Indeed, applications of self-determination theory in professional development reveal that educators who feel autonomous, competent, and connected are more likely to engage and foster their personal and professional growth (39). This notion aligns with a self-directed approach towards teaching which encourages instructors to acknowledge, exercise and experience professional autonomy. By exploring and implementing various teaching strategies that align with their professional goals, educators can nurture their intrinsic motivation and enhance their teaching effectiveness. Moreover, educators would focus on building their teaching competencies through ongoing professional development, for instance, by seeking out challenging learning opportunities, engaging in reflective practice, and utilizing feedback to improve their skills.

Constructivist theory, rooted in the work of Piaget (40) and Vygotsky (41) has most commonly been defined as a way to understand how learners construct their own understanding and knowledge through experiences and reflection. Accordingly, learning is an active and contextualized process of constructing meaning, rather than passively receiving and processing information. This notion implies that learners have control over their learning processes. By formalizing the role of instructors’ autonomy in the educational process, SDT synthesizes and extends the principles of constructivism. While constructivist theory highlights the importance of learners’ active role in their own learning journey, SDT encourages educators to design teaching experiences that give them greater control over their teaching activities and strategies.

Furthermore, constructivist learning strategies, such as scaffolding—where temporary support is provided to learners to help them achieve a deeper understanding or skill- align well with SDT. Educators can utilize a scaffolding approach in their professional development by initially seeking guidance and feedback from mentors and professional networks or initiating peer evaluations. As educators become more self-directed, collaborate with colleagues they take greater ownership of their development, adjusting their teaching methods based on ongoing reflection and feedback. This process ensures that professional growth is both continuous and responsive to the shifting demands of the educational environment. Furthermore, collaborative learning, another key approach of constructivist theory, is essential for SDT. Educators can gain new perspectives and develop new teaching skills by actively participating in professional learning communities, collaborating with colleagues on teaching projects, and engage in reflective discussions pertaining to their own teaching skills and experiences.

In conjunction, these theories provide a robust theoretical foundation for self-directed teaching. Consequently, SDT may be discerned as a contemporary application of these theories.

## 4 Key components of self-directed teaching

**Figure 1:**
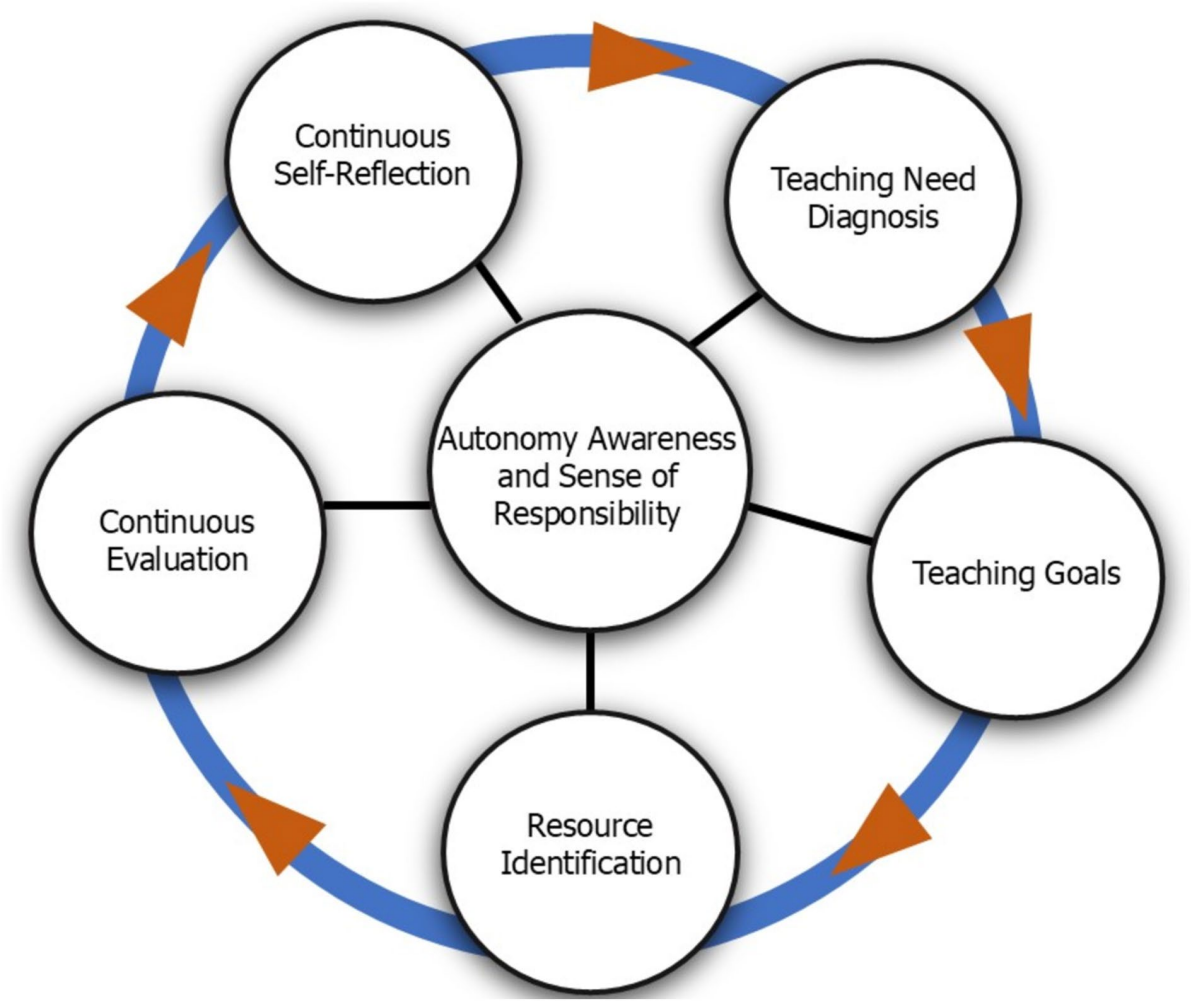
This figure illustrates the key components of self-directed teaching (SDT), with autonomy awareness and sense of responsibility at the center, serving as the foundation of the framework. The arrows along the blue line highlight the ongoing, cyclical nature of the process, emphasizing that these components are not only interconnected but also continually influence each other. The components include continuous self-reflection, which involves regularly reflecting on teaching methods and experiences to foster growth and improvement; teaching need diagnosis, referring to identifying areas where teaching practices require improvement or adjustment to better meet student needs; teaching goals, involving the establishment of clear, achievable objectives aligned with diagnosed needs; resource identification, the process of finding and utilizing resources essential for reaching the set goals; and continuous evaluation, which entails ongoing assessment of teaching practices and goals to ensure progress and make necessary adjustments. Together, these components work dynamically to support the continuous development and refinement of self-directed teaching practices.

In the following (see Figure 1), the specific components that constitute and form the core of SDT are introduced.

### 4.1 Autonomy awareness and sense of responsibility

Deci and Ryan (42) highlight that autonomy awareness fosters the intrinsic motivation necessary for intentional and goal-directed behavior and is a fundamental prerequisite for self-determination. Consequently, understanding and recognizing one’s autonomy strength the motivation to pursue goals and is essential for forming and pursuing any type of self-directed behavior (43). Therefore, the first step toward self-directed teaching should be gaining the awareness of one’s autonomy to take charge of one’s teaching. Evidence provided from research on the intersection between self-directed learning and self-determination theory supports this notion. Studies have shown that a key reason why many students struggle to adapt to SDL (44) and feel overwhelmed by the autonomy it requires (31, 45), is they lack autonomy awareness (45). These findings allow us to assume that without first developing autonomy awareness, educators may not adhere to SDT. Therefore, ensuring that educators recognize and embrace their autonomy is a crucial first step in successful implementation of SDT.

Evidence-based strategies to gain autonomy awareness:

1. Reflective practices: Empirical evidence suggests that reflective practices can promote awareness of own professional autonomy and is a powerful tool for teachers to gain a better understanding of their professional actions and responsibilities (46, 47).
2. Self-regulation training: Training in self-regulation has been shown to enhance autonomy awareness (15, 48–50). In a related vein, Zimmerman (44) reported that training in self-regulation helps students to effectively engage in self-directed learning.
3. Collegial learning activities (CLAs): CLAs can provide a supportive ground to evaluate goals and plans, share teaching experiences, reflect on teaching practices, and receive feedback from peers. This collaborative approach can help educators to recognize their autonomy and discover feasible approaches to effectively exercise it. For instance, Stoll et al. (51) report that this type of activity enhances collaborative learning and reflective dialogue, and hence, is crucial for developing autonomy awareness and self-direction in teaching.

### 4.2 Teaching needs diagnosis

The next step toward self-directed teaching is to identify areas where educators need to improve their knowledge, skills, and methods. It also pertains to establish which new skills must be required to be able to respond to students’ diverse learning needs. Tools such as student feedback, peer reviews, and self-reflection are used to diagnose these needs.

Evidence-based strategies for teaching needs diagnosis:

1. Self-reflection: Building on the role of self-reflection discussed in previous section, engaging in regular self-reflection allows educators to critically examine their teaching practices, methods, and interactions with students. Through this introspective process, educators can identify areas where their teaching may fall short and recognize the need to enhance existing skills or the necessity for enquiring new ones to better meet their students’ learning needs.
2. Student feedback: It seems self-evident to claim that students can teach us how to be good teachers. Feedback from students through teaching evaluation surveys, interviews, and informal conversations can provide valuable insights into how teaching practices affect their learning and help to establish areas in need of improvement (52). For instance, recently was reported that incorporating students’ feedback in the process of development and revision of exam questions can significantly enhance the quality of exams (85).
3. Peer observation and peer review: Fellow teachers can provide critical insight and perspectives on all relevant aspects of teaching. Therefore, participating in peer observations and reviews can enhance professional development (53).
4. Performance data analysis: Student performance data (e.g., test scores, attendance rates) can help to identify where teaching strategies need adjustment (54).

### 4.3 Goal setting and goal attainment

#### 4.3.1 Goal setting

Self-directed implies that the teaching approach is directed towards achieving certain goals. Once teaching needs have been established, educators must define goals that are specific, measurable, and feasible to address their identified needs (49). However, it has long been recommended that is crucial to differentiate between short-term and long-term goals. Short-term goals help educators achieve immediate improvements and recognition while working towards broader long-term objectives. For example, a short-term goal might be to increase student engagement (measured by the frequency of active participation in class debates) over the next 3 months by incorporating certain interactive activities in the classroom. Whereas a long-term goal might be to pursue an advanced degree in instructional design in the next 5 years.

#### 4.3.2 Goal attainment

An effective goal attainment requires detailed action plans for each short- or long-term goal to guide educator’s actions. Action plans should be detailed and include specific activities and identify resources that are needed. In addition, they should also anticipate potential obstacles and challenges (44).

### 4.4 Resource identification

Resource identification pertains capitalizing on available resources and, if needed, pursuing additional resources to ascertain efficient goal attainment (55).

Evidence-based strategies for resource identification:

1. Professional development programs: Enrolling in professional development (e.g., courses, workshops, and seminars) provide structured learning opportunities to gain new knowledge and skill (56).
2. Educational literature can provide new insights on evidence-based approaches, technical advancements and tools and stay informed with most recent developments and trends in education (8, 57).
3. Mentorship and collaboration: Seeking mentorship from experienced educators and collaborating with peers can provide support, guidance, and new perspectives (58).

### 4.5 Continuous evaluation

Sections 4.1–4.4—autonomy awareness, teaching needs diagnosis, goal setting, and resource identification—naturally culminate in the revision of current teaching strategies or the implementation of new ones. This process does not end with the application of new methods; rather, it requires continuous assessment, reflection, and adjustments to ensure their quality and impact. This ongoing cycle allows educators to refine or set new goals and plans for future professional development. Goals that are deemed to be unattainable or infeasible can be adjusted or replaced. For instance, if pursuing an advanced degree in instructional design in the next 5 years turns to be infeasible, e.g., due to financial burden, it could be adjusted to pursue a certificate in instructional design instead. This cyclical process of goal setting, regular monitoring, and adjustments are essential to maintaining progress.

Strategies for evaluation:

1. Pre-post peer evaluation: Engaging in peer evaluations before and after implementing new strategies allow to evaluate their quality, and impact and identify areas for improvement.
2. Pre-post comparisons of student performance data: Utilizing formative (e.g., quizzes, peer reviews) and summative assessments (e.g., final exams, projects) both before and after implementing new strategies provides comprehensive data on student learning outcomes. This evidence-based approach enables educators to measure the effectiveness of their teaching methods and make informed adjustments (54). Advanced analytic approaches, such as piecewise latent growth modeling (LGM), can further quantify and accurately capture reliable changes in student performance offering a more nuanced understanding of the impact of educational interventions (59, 60).
3. Pre-post comparisons of student feedback and teaching evaluations: Regularly collecting and analyzing student feedback before and after changes are made helps educators gain insight into students’ perceptions and identify areas for improvement. Methods for gathering feedback can include surveys, focus groups, and informal discussions, which collectively offer a well-rounded understanding of the student experience (61).

### 4.6 Continues self-reflection

Self-reflection is a critical process that allows educators to evaluate their teaching practices, identify areas for growth, and foster continuous improvement (46, 62). One effective tool for this ongoing process is a self-reflection journal. By regularly documenting experiences and recording reflections, educators can create a personal record to track their growth over time and assess whether their teaching approach align with their goals (63). Additionally, these journals can be valuable resources for guiding and mentoring fellow educators who are interested in pursuing a self-directed teaching approach.

## 5 Significance of self-directed teaching

By applying these principles, educators can address several critical issues, including:

1. Professional autonomy and intrinsic motivation: SDT is grounded on and at the same time fosters a sense of autonomy and responsibility over professional development, which can significantly enhance motivation and job satisfaction among educators (64). When educators recognize and acknowledge their autonomy to define and pursue their teaching related goals, they are better equipped to face professional challenges (39) and are more likely to feel committed to continuous improvement (65, 79).
2. Adaptability to diverse learning needs: Educational standards and societal definition of and expectations from adequate and efficient teaching is constantly advancing (7, 31). Consequently, teachers must be capable to tailor their teaching to address diverse needs of their students. SDT encourages educators to move beyond traditional teaching approaches and acknowledge the unique learning styles of their students and adopt flexibly and personalize their teaching approaches (66). For instance, continuous self-assessment and reflection, key components of SDT, enables educators to timely identify critical areas where their students require different teaching approaches or additional support and adjust their teaching methods to better meet the needs of students (8). Students who may struggle with traditional teaching methods, might learn better if visual aids, interactive tools, or differentiated instruction are incorporating in the teaching materials.
3. Integration of technology: The implementation and effective integration of technology in the classroom is not a temporary or reversible trend. It is a programmatic and paradigmatic change in education (67). Adhering to a SDT approach enables and encourages educators to assess their personal needs to enhance their digital literacy and seek out and experiment with new technological tools and resources and incorporate technology in meaningful ways (57). Moreover, staying current with technological innovations, such as digital learning platforms and adaptive learning technologies, enables educators to create more engaging and interactive learning environments, further enhancing the effectiveness of their instruction (68).
4. Staying current with pedagogical advances: Educational research is exponentially growing and continually offering new insights into how to enhance students’ engagement and learning outcomes (69). SDT motivates educators to welcome these developments and integrate evidence-based practices into their teaching and ensure that their teaching methods remain relevant and effective in promoting student learning (70). For instance, educational research has shown that evidence-based practices, such as formative assessments, collaborative learning, and feedback-driven instruction, significantly impact student achievement and engagement (71). SDT encourages educators to stay informed about these advancements through continuous professional development, reflective practices, and active participation in professional learning communities (7). By incorporating the latest pedagogical research into their teaching, educators can adapt their methods to address diverse learning needs, promote deeper learning, and improve overall student outcomes. This commitment to staying up-to-date ensures that teaching practices remain relevant, responsive, and grounded in the latest research, ultimately fostering better learning experiences for students.
5. Lifelong learning for educators: Just as the zeitgeist encourages students to be lifelong learners, educators must also embody this principle (78). SDT aligns perfectly with the ethos of lifelong learning by fostering a culture of continuous professional development and reflective practice. Through SDT, educators take ownership of their professional growth, regularly seeking out new knowledge, teaching strategies, and technological advancements to refine their practices (72). This process of continuous inquiry not only enhances individual teaching effectiveness but also strengthens the broader educational system by ensuring that educators remain adaptable and capable of addressing emerging challenges in education. Lifelong learning in SDT involves participating in professional learning communities, pursuing advanced certifications, engaging in research, and embracing feedback to improve instructional methods (73). Moreover, by adopting a lifelong learning approach, educators model the importance of ongoing development for their students, creating a learning culture that values persistence, growth, and adaptability (74). This commitment to lifelong learning ultimately benefits both educators and students, contributing to a more dynamic, innovative, and responsive educational environment.
6. Addressing teacher burnout: The teaching profession is demanding and stressful. Educators report high levels of burnout (75, 76, 81). SDT can help mitigate these issues. By exercising their autonomy, setting their own goals, proactively pursuing and tailoring their own professional growth educators can find renewed purpose and satisfaction in their careers and reduce their vulnerability for burnout (77).

## 6 Conclusion and future directions

Self-directed teaching (SDT) is proposed as a holistic theory-based framework that integrates multiple core aspects of the teaching process into a cohesive framework. SDT integrates principles from educational theories such as self-directed learning, self-determination theory, and constructivism, offering an empowering, holistic, and proactive approach to addressing the complexities and challenges of modern education. By encouraging educators to engage in reflective practice, diagnose their teaching needs, set personal goals, and continuously refine their methods, SDT provides a pathway for enhancing teaching effectiveness for students and professional satisfaction for educators. The framework’s adaptability allows educators to meet diverse student needs, stay current with pedagogical advances, and promote lifelong learning, ultimately leading to more meaningful and fulfilling teaching experiences. Through its potential to reduce burnout, increase teacher motivation, and improve student outcomes, SDT offers a valuable approach to modernizing teaching practices in a rapidly evolving educational landscape. Further research and practical applications of SDT will be crucial in optimizing its implementation and understanding its broader impact on education.

Further research and theoretical development are needed to bring SDT from a conceptual framework to a practical approach, to understand its impact and optimize its implementation. For instance, pilot programs in diverse educational settings using both qualitative and quantitative measures, such as educator self-reports, peer evaluations, and student feedback are needed to establish the feasibility of SDT and its associations with variables such as teaching evaluations, students’ performance, burnout, and job satisfaction. In addition, longitudinal studies can provide insight to establish the long-term impact of SDT on these variables.

Research into the application of SDT across diverse educational contexts—including various grade levels, subject areas, and cultural settings—will help tailor these approaches to different educational environments and student’s needs. In addition, the impact of mentoring and peer support on the effectiveness of SDT should be investigated, with a focus on identifying best practices for supporting emerging educators. Lastly, examining how institutional policies can better support SDT is crucial including how SDT principles can be integrated into teaching and teachers’ evaluation systems, professional development programs, and curriculum revisions and improvement plans.

By addressing these areas, educators can refine and enhance self-directed teaching, leading to more effective and fulfilling educational experiences for both educators and students.

## Data Availability

The original contributions presented in the study are included in the article/supplementary material, further inquiries can be directed to the corresponding author.
